# Pain Management in People With Dementia Living in Nursing Homes: A Focused Ethnographic Study

**DOI:** 10.1177/14713012251358003

**Published:** 2025-07-03

**Authors:** Caroline Kreppen Overen, Siren Eriksen, Maria Larsson, Adelheid Hummelvoll Hillestad

**Affiliations:** 1 155319 Lovisenberg Diaconal University College, Oslo, Norway; 2 Faculty of Health, Science and Technology, Department of Health Sciences, 4209 Karlstad University, Karlstad, Sweden

**Keywords:** dementia, nursing home, palliative care, pain management, pain assessment, person-centred care

## Abstract

The literature on pain management for people with dementia in nursing homes places significant emphasis on the limitations dementia imposes on their ability to describe and express their pain. Little is known about how healthcare personnel can support and use the individual resources people with dementia have in pain management. Thus, this study aimed to explore pain management in people with dementia in nursing homes, focusing on the resources people with dementia have and how healthcare personnel can support and integrate these resources into their clinical practice. We applied an exploratory design with a focused ethnographic approach and collected data through participatory observations and field conversations, as well as notes from residents’ medical charts. The study setting was a nursing home unit specializing in dementia care in a larger city in Norway. The study included residents with moderate- to severe stage dementia (*n* = 18) and nurses caring for them (*n* = 12). The data were analysed in line with qualitative content analysis. The analysis identified two categories: (a) Residents’ pain narratives and (b) Nurses’ interpretation and support. Within each category, several subcategories were identified. The categories are linked together in one overarching, latent theme describing how people with dementia express themselves in the moment and nurses integrate these moments into a coherent whole. Findings also indicate that people with dementia have individual resources and strengths that are important for pain management. With support from healthcare personnel, they can be active participants in pain management processes, in accordance with their individual abilities.

## Introduction

Pain in people with dementia is associated with reduced quality of life (Helvik et al., 2022), and up to 80% of people with dementia living in nursing homes experience pain on a regular basis (Helvik et al., 2023). Untreated pain can lead to reduced physical function (Plooij et al., 2012), decline in activity of daily living function (van Dalen-Kok et al., 2021) and depression (Malara et al., 2016). This underscores the importance of pain management for the target group (Radbruch et al., 2020; van der Steen et al., 2014). Moderate to severe stages of dementia are associated with declining physical and cognitive function, as well as loss of autonomy and independence (Hanson et al., 2019). Therefore, a palliative care approach is considered appropriate throughout the dementia disease trajectory (van der Steen et al., 2014). A main goal of palliative care is quality of life (Radbruch et al., 2020), for which effective symptom control, including management of pain, is an important predictor (Ojelabi et al., 2017).

The assessment and reporting of pain in people with dementia are widely recognized as a complex challenge across settings. Healthcare personnel often express uncertainty regarding the most effective strategies to support people with dementia who experience pain (Smith et al., 2023). The subjective experience is essential in pain management (Wideman et al., 2019), but people with dementia often have difficulty expressing their subjective experience of pain (Sirsch et al., 2020; Woodward, 2013). Some express pain in the form of psychological symptoms or changed behaviour, such as restlessness or withdrawing (Atee et al., 2021). While these symptoms and behaviour changes may also have causes other than pain, it is important for healthcare personnel to know what constitute ‘normal’ behaviours of people with dementia (Smith et al., 2023).

Most people with dementia will need nursing home care (Wergeland et al., 2015). Nursing homes are complex institutions that function as a home for residents in need of extensive and comprehensive treatment and care (Fassmer et al., 2024; Schweighart et al., 2022; van den Brink et al., 2018; Ågotnes et al., 2019) and as a workplace for healthcare personnel (Rokstad et al., 2023; Vermeerbergen et al., 2017). Globally, the prevalence of people with dementia in nursing homes differs by location, country and region (Seitz et al., 2010), and over 83% of the Norwegian nursing home residents have dementia (Gjøra et al., 2021). In this population, behavioural and psychological symptoms are common, with a reported prevalence of 56%–70% (Helvik et al., 2018). Most residents with dementia will have additional medical conditions potentially causing pain (Browne et al., 2017). In Norwegian nursing homes, it is primarily nurses, in collaboration with other professional groups and relatives, who provide daily care, suspect and/or detect pain, and initiate pain assessment.

Smith et al. (2023) emphasize that pain should be managed via a person-centred approach, in which respecting the person’s individual abilities and resources are core values (McCormack et al., 2021). In pain management of people with dementia these resources can be seen as remaining verbal communication skills and non-verbal embodied communication. Hydèn (2011, 2014) discusses how people with dementia can participate, for example through telling a story, gaining access to the communicative and cognitive resources in interactions and collaboration with others. Kontos (2004) argues the importance of embodiment and how people with dementia communicate through their body. Communication of pain through body language is recognized and widely explored and often serves as a first indication of pain in people with dementia (Overen et al., 2025). An understanding of the resources of people with dementia in pain management is a direct consequence of how we understand personhood. Boddington et al. (2024) emphasizes embodied and relational personhood, while simultaneously advocating a need to retain recognition of more cognitive and rational approaches; that multiple conceptions of personhood might enable elements of a rational and cognitive description of personhood to “flourish”. Nurses experience difficulties in finding the underlying cause of changed behaviour, and there often is great uncertainty about the subjective experience of pain. A broad perspective on resources of people with dementia is needed and lacking in pain research (Overen et al., 2025). Thus, this study aimed to explore pain management in people with dementia living in nursing homes, focusing on the resources people with dementia have and how healthcare personnel can support and integrate these resources into their clinical practice.

The following research questions guided the study:

(1) How do people with dementia express their subjective experience of pain, and how can they be supported in expressing their experience?
(2) How do nurses use these expressions and integrate residents’ resources in pain management?
(3) How does the care environment influence the integration of residents’ resources in pain management?

## Methods

We applied an exploratory design with a focused ethnographic approach (Higginbottom et al., 2013). Utilizing this approach, we gained an in-depth perspective on the interactions between people with dementia and nurses around pain management, within the context of nursing homes. Ethnography is a well-recognized, appropriate and valid method in dementia research (Featherstone et al., 2022; Kelley et al., 2021). Ethnography adopts an epistemological approach that acknowledges the importance of embodied and relational aspects of personhood, and enabled us to capture data that could not be verbalized (Boddington et al., 2024). The preparation of this paper adhered to the Standard for Reporting Qualitative Research (SRQR) guidelines (O’Brien et al., 2014).

### Setting

Approximately 30% of the nursing home units in Norway are specially adapted for people with dementia (Norwegian Directorate of Health, 2019). The unit in which this study was conducted was in a larger city in Norway and constituted 17 single rooms with private bathrooms. The residents could bring their personal belongings, including furniture. The rooms were divided into two groups, and each group had an associated common area consisting of a living room, kitchen and veranda. In the middle of a hallway that separated the groups, there was a staff office; this was connected to a medication room and the manager’s office. The unit was staffed by certified nursing assistants and registered nurses, as well as a smaller number of healthcare assistants. A typical weekday day shift was staffed with three certified nursing assistants or healthcare assistants, one registered nurse, one nursing development specialist (registered nurse) and the unit manager (registered nurse). Physicians were available every weekday during office hours, as were an occupational therapist, physiotherapist and activities coordinator.

### Recruitment Strategy and Participants

Contact was established with the senior quality manager at the nursing home, who shared information about the study with the unit manager. The first author (CKO) presented information about the study at a staff meeting. The unit manager conveyed written and verbal information about the study to potential participants and their relatives and collected consent forms. An assessment of competence to consent was carried out by a registered nurse who knew the residents well. For residents who lacked the capacity to provide consent, proxy consent was obtained from a relative. A total of 30 people agreed to participate in the study, including 18 people with dementia and 12 nurses. The nurses included 7 certified nursing assistants and 5 registered nurses. All the residents lived on the ward due to care needs related to their diagnosis of moderate- to severe stage dementia. Three residents passed away during data collection, so two additional residents were included in the study upon their admission to the unit. Characteristics of the participants are presented in Table 1.

**Table 1.** Participant Characteristics

Residents with dementia (*n* = 18)	Age	Mean = 84 years (min 57–max 101)
	Gender	Women (*n* = 12)
		Men (*n* = 6)
	Dementia diagnosis	Alzheimer’s dementia (*n* = 6)
		Frontotemporal dementia (*n* = 1)
		Lewy body dementia (*n* = 1)
		Mixed dementia (*n* = 1)
		Unspecified dementia^a^ (*n* = 9)
	Length of nursing home stay	Mean = 2.5 years (min 1 month–max 6 years)
Nurses (*n* = 12)	Age^b^	Mean = 45 years (min 21–max 61)
	Gender	Women (*n* = 11)
		Men (*n* = 1)
	Authorization	Certified nursing assistants (*n* = 7)
		Registered nurses (*n* = 5)
		Of these, 5 CNAs and 3 RNs had courses and/or education in dementia or palliative care after basic education.
	Length of time working with people with dementia^b^	Mean = 13 years (min 2.5–max 27)
	Length of time working at the specific unit^b^	Mean = 7 years (min 6 months–max 16 years)

^a^Unspecified dementia is prevalent in Norway, and poses significant challenges (Gjøra et al., 2024).

^b^Data from one participant is missing.

### Data Collection

Data were collected by CKO between September 2023 and February 2024. Higginbottom et al. (2013) emphasize that an ethnographic approach requires multiple data collection methods to achieve a sufficient depth of understanding. Accordingly, the collected data material consisted of descriptive and reflexive notes from participant observations, notes from conversations with residents and nurses that took place during fieldwork, and notes from residents’ medical charts, to which access was granted through the informed consent. Data were collected until saturation was reached.

To avoid giving residents the impression that she was one of the ward nurses – and to maintain a clear personal distinction between her nursing role and her role as a researcher – CKO wore her own clothes, rather than a uniform. An explorative observation protocol separated descriptive and reflexive notes and facilitated a structured mapping of ‘pain episodes’, including the time, context, people involved and source of information (i.e., direct observation, conversations while in the field or medical chart note). The descriptive notes provided detailed and objective accounts of the observations. The reflective notes were more subjective, and included CKO thoughts, experiences, questions and interpretations of the observations.

Observations took place over 182 h, distributed over 29 days with an average duration of 6.25 hours per day (min 1.5 h – max 8.5 h) during both day and evening shifts. Initially, different times were prioritized to ensure sufficient variation, but as data collection progressed, the observations took place at times considered most relevant to the study’s focus – i.e., during morning care routines and oral reports. CKO was present in everyday activities at the nursing home unit, with the aim of observing a wide range of different care situations. The nursing home unit context made residents’ ‘pain episodes’ readily observable, making it possible to identify and follow multiple episodes in different residents over time.

### Data Analysis

The analysis began during the observation period and continued throughout the writing and revising of the presentation of the results (Holloway, 2021). In line with Higginbottom et al. (2013), this was an iterative, cyclic and self-reflexive process that involved challenging preliminary interpretations, revisiting earlier data and planning how to generate new insights into the data. To classify and explain patterns, as described by Higginbottom et al. (2013), we adopted Graneheim and Lundman’s (2004) approach to content analysis. This included both their manifest and latent approach, which is further elaborated upon by Lindgren et al. (2020). The analysis was conducted in five steps (Graneheim & Lundman, 2004): (1) All field notes were read in their entirety and discussed by two researchers (CKO and AHH), and (2) meaning units in the data material were extracted by CKO. (3) The meaning units were coded using NVivo (Jackson et al., 2019); the initial coding was carried out by CKO, and then discussed and further developed in close collaboration with AHH. (4) The codes were organized into subcategories and abstracted into three main categories describing the manifest level of the results. (5) The categories and their corresponding subcategories were then reflected upon to identify the latent meaning of the results, resulting in the identification of an overarching theme. All authors were involved in steps 4 and 5.

### Preunderstanding

Higginbottom et al. (2013) emphasize reflexivity in ethnographic research, with preunderstanding as an essential aspect. The observer (CKO) is a nurse practitioner specialized in geriatrics and palliative care, with extensive experience working as a nurse in care units for people with dementia. Prior to the observations, the second author (SE) interviewed CKO about her experiences working with palliative care for people with dementia. This interview highlighted and promoted awareness of her previous experiences and preunderstandings – necessary prerequisites for understanding the complex phenomenon of pain management in people with dementia. The three co-authors are Registered Nurses with clinical experience in palliative and geriatric care, and they are all experienced in qualitative research. Two of them (AHH and SE) have extensive knowledge about dementia and nursing homes as a context, and one (ML) has extensive knowledge about palliative care and symptom management. Through the exploration of pain management, our understanding of the phenomena was developed by going back and forth in the process of data collection, analysis and writing, with an openness to developing and changing this understanding.

## Results

The analysis identified two categories: (a) residents’ pain narratives and (b) nurses’ interpretation and support. Both categories contained several subcategories, as shown in Table 2. The categories are linked together in one overarching, latent theme. In this results section, the term ‘nurse’ encompasses both certified nursing assistants and registered nurses. Where relevant, we distinguish between these roles.

**Table 2.** Identified Subcategories, Categories and Theme

Subcategory	Category	Overarching theme
The importance of a safe care environment	Residents’ pain narratives	The residents express themselves in the moment and nurses integrate these moments into a coherent whole
Expressions of pain		
Different dimensions of pain		
Influence of care environment	Nurses’ interpretation and support	
Encouraging and facilitating self-reporting		
Using the residents’ pain expressions		
Clinical competence		

### Residents’ Pain Narratives

The observations revealed that residents experienced moments of pain and carried these moments with them, as fragments, but had difficulty gathering these fragments together into a cohesive pain narrative. This category highlights the residents’ prerequisites for being able to contribute to the creation and maintenance of a pain narrative. It consists of three subcategories: (a) the importance of a safe care environment, (b) expressions of pain and (c) different dimensions of pain.

#### The Importance of a Safe Care Environment

The context in which the residents’ perceptions and expressions of pain took place was dynamic. There were different people at work, periods with students and relatives present, and each day had its own schedule. Residents’ health conditions were also changeable, as was their perception of time, place and the people present. Residents also experienced delusions. Despite the dynamic context, the unit facilitated a safe, stable and predictable environment with established routines – including flexibility, where the nurses tailored their care approach to residents’ expressed priorities and boundaries. When talking about pain, several of the residents highlighted the importance of feeling safe, and how the unit constituted a safe place where they could ‘be themselves’ and express their experiences:

I’ve been at rock bottom. Then life was very difficult. But here it is so much better; here I am safe. They are so good here. The people who work here become like your own. They look after you. It’s so safe here – here we can be ourselves, for better or worse. Here, we are all equal.

#### Expressions of Pain

The residents could understand and describe situations when they had experienced pain, but their fluctuating perceptions of time and space meant that their accounts sometimes differed from what the nurses observed. For example, after falling out of bed, one resident described the experience as lying under a fishing pier and calling for help for several hours. The nurses understood the situation as a fall without visible injuries, noting that the resident did not express pain in any way. Nonetheless, how the resident expressed his experience constituted an important aspect of how this situation affected him – possibly also evoking previous experiences from his life.

The residents expressed pain in several ways, one of which was indirectly asking for help. In one instance, a nurse entered a resident’s room when she called for her mum. When asked whether she was in pain, the resident answered that she had a headache. In general, during the study period, there was a high frequency of self-reporting by the residents – both spontaneous and encouraged. The nurses emphasized that ‘ability to self-report’ fluctuated, and if the resident felt stressed, it could negatively affect their ability to self-report.

Non-verbal embodied communication potentially related to pain was frequently observed in the unit. Examples include residents walking restlessly in the corridor appearing uncomfortable or stressed, or residents withdrawing to their own room. Equally important was the opposite, where the residents appeared comfortable and content with a relaxed facial expression.

Documentation of change from normal state: The resident is perceived as tired and unmotivated, takes little to no initiative to talk, or asking for assistance.

Table 3 provides examples of the different types of pain expressions that were observed.

**Table 3.** Examples of the Residents’ Pain Expressions

Type of pain expression	Data excerpt
Spontaneous self-reporting	I have such a headache. It’s pressing. Here (puts her hand on forehead), and down here (stroking right side of her face). Aaaaaw, it hurts so much. In my ear, my ear hurts so much. I don’t understand what is happening to me. My body is so weird. I don’t eat, I don’t feel like... Oouh, you must help me.
Encouraged self-reporting	Nurse to resident: Tell the doctor how you feel, how it feels. Resident: It’s burning! Wound infection. I had ulcers. Someone said that. It’s an infection, my leg is throbbing!
Spontaneous pain words	The resident says ‘auauauauau’ when the syringe is inserted
Pain behaviour with strong suggestion of pain	The resident holds her head in her hands, leans forward and rubs her temples
General behavioural change	The resident stopped wandering down the corridor as he usually does

The residents had different starting points, prerequisites, and abilities to contextualize their pain. Previous experiences, expectations and insights into their own situation – as well as their understanding of the context of that pain and their individual stories about previous experienced pain – all contributed to how they talked about and expressed pain.

CKO: Are you in pain?

Resident: It depends on what you mean by pain. It could be in the stomach or in the head.

CKO: Do you have pain there, in your stomach or in your head?

Resident: I’ve been to the hospital a few times over the years, with pain. I don’t know where they come from, the pain in my head. I have pain when there is something wrong with my body. My body is so weird, I don’t understand anything.

The residents also had expectations around potentially painful situations. Visible conditions that were painful (e.g., a wound or rash) or situations where pain could clearly be expected, promoted spontaneous self-reporting. A wound being dressed is an example of one such situation.

The resident sits in a chair in the living room, with her leg on an ottoman. The nurse is changing a wound dressing on the resident’s leg. The nurse removes the bandage. Resident says, ‘Imagine that I got a wound right there, on my calf’. The nurse applies saline compresses. The nurse then looks up at the resident’s face – says nothing. Resident then says, ‘It doesn't hurt’.

#### Different Dimensions of Pain

The residents described an understanding of pain as having multiple dimensions, including psychological, social, physical and existential. When asked what pain was, from her perspective, one resident described it as social and existential. She expressed pain at missing out, longing for visits from her loved ones and not knowing about the future:

There’s a lot I can’t take part in. Whether I’m too old or too young, not everything is suitable for all ages. I belong, but none of mine are here. I sit and wait; they are supposed to come. It’s no fun sitting here and waiting when I don’t know what’s going to happen.

Another resident also described her perception of pain as multidimensional, drawing from previous experiences in both her professional and private life. She related it to her own past experiences with pain, as well as the pain she had observed in others:

Resident: I don’t have any pain anywhere – it was worse before, but now it’s fine. (...) Now I know what I get, and I also know that the people who work here are doing the best they can. I have worked with children who were in pain, who had parents who were not kind to them.

CKO: (…) Do you think that pain is about something more than just having pain in the body?

Resident: Yes, absolutely. If you have a hard time, it hurts (holds her hands over her chest). For example, if you think about something that you have done, whether it was right or wrong.

### Nurses’ Interpretation and Support

We found that the nurses who had been present for an extended period of time were able to support residents in self-reporting by drawing on their knowledge of time, space and the residents’ pain history. The nurses acted on, interpreted and communicated the residents’ expressions of pain, regardless of type. This category consists of four subcategories: (a) influence of care environment, (b) encouraging and facilitating self-reporting, (c) using the residents’ pain expressions and (d) clinical competence.

#### Influence of Care Environment

The nurses constantly needed to adapt to wide variations in what constituted typical behaviour for each resident, as well as in how they expressed and talked about their pain. For some residents, personal care situations were observed to be a suitable setting for the nurses to map and assess physical pain, as these interactions included both guided and spontaneous movements. For instance, the nurses could observe a resident’s skin in a setting where it was natural for the resident to undress. However, personal care situations were also observed to be a potential cause of major distress for the residents. When residents had reduced language and ability to self-report pain, it was challenging for the nurses to distinguish between different causes of general pain behaviour in contexts the residents found stressful. An expression of pain could reflect that a resident was feeling uncomfortable in the situation, rather than feeling physical pain.

In a personal care situation, the resident shouts “no, no, no”, loudly and intensely when lifting the arms

Nurse: “Do you have pain”?

Resident: “I don’t know what I have”

Nurse (with an empathetic voice): You don’t know what you have, *name of resident*, are you scared?

Resident: Yes, I’m scared. I’m so scared, so scared, so scared.

The nurses take several breaks when the resident starts shouting. Caresses her forehead, says “we’re here, we’re taking care of you”

The nursing home had an explicit person-centred culture that focused on the residents’ perspectives, preferences and experience of the situation.

Nurse: She (a resident) kept saying “let me sit in peace and quiet”. She kind of blocked everything out. She could gradually let in a little bit at a time. That autonomy, that self-determination... She said she was in pain and was offered a morphine injection. But when we came with the syringe. Then that injection was much worse than the pain. She got the offer but decided for herself. She wasn't going to have an injection (she was offered another administration of painkillers, which she accepted). That self-determination is important.

In order to meet the rapidly changing needs of each resident and the wide variations, the nurses emphasized interdisciplinary collaboration and competence-enhancing measures anchored within the leadership of the nursing home.

#### Encouraging and Facilitating Self-Reporting

Familiarity with the residents’ normal state enabled nurses to encourage residents in self-reporting if a change was noticed. The nurses were also observed to promote self-reporting and the residents’ participation by optimizing communication, for example with appropriate use of hearing aids and glasses or assessing their pain in quiet surroundings, in a room the resident experienced as safe. The nurses also made use of supportive communication, enabling the resident to share their experiences with colleagues:

The resident says that ‘it hurts on the bump’ (her knuckle), and ‘in-between’. She points with her fingers around the wrist and says, ‘It’s a pain there and there’. The nurse replies, ‘You know what, we have to take this to the Registered Nurse’. They get up and walk together down the corridor. The nurse says, ‘Now you have to tell *name of the registered nurse* what you told me, that you were in pain’. The nurse carefully guides the resident’s arm up towards the registered nurse. The resident says that ‘my hand hurts like hell’ and ‘anyone who gets close to it gets a slap in the face’.

#### Using the Residents’ Pain Expressions

Our findings indicate that self-reporting and residual resources in people with dementia were often integrated into pain management processes. The residents’ descriptions of pain could be included as a part of the larger whole, combined with supplemental information. For example, one resident often expressed having neck pain. However, nurses directed measures towards the environment and the resident’s sense of safety, because they had observed that she did not express neck pain when there were nurses in the immediate vicinity. They knew that, before proceeding to pharmacological interventions, they could try simply sitting down with her when she expressed pain.

The nurses employed several different approaches when they suspected and/or discovered that a resident had pain. They based their approach on verbal pain expressions from the residents or on pain behaviour (such as touching or rubbing an area of their body). In situations where there was a direct and/or visible cause of pain or a condition where pain was expected (‘If you have a broken hip, you are in pain’) contrasted with more ambiguous cases, where the nurses were unsure whether the resident was experiencing pain. Acknowledging that they could never be completely certain, they employed various strategies to confirm or dismiss their suspicions. Notably, there was significant interpretation involved in cases where pain expressions were absent.

Nurse: Are you in pain?

Resident: Yes. I have no legs to walk on.

Nurse: Is it the legs that hurt?

Resident: Yes, they are what they are.

The nurse asks if she wants to walk with her. The resident gets up without an expression of pain, and they walk together down the corridor. The nurse asks again, ‘Do you have any pain?’

Resident: No, not right now, now it’s fine. But earlier I wasn’t sure if I should answer yes or no. It is not always easy to know what the right answer is.

When asked about whether she was experiencing pain, the resident answered that she was not, when walking with the nurse. The nurse observed no expressions of pain during guided movement and a specific activity. The nurse subsequently concluded that the resident was not in pain anymore.

#### Clinical Competence

The nurses’ interpretation of residents’ expressions of pain was influenced by their clinical competence, including their expectations regarding which conditions are typically painful, their clinical observations and assessments and their knowledge of which examinations could be carried out to detect pain. They used several clinical observations and assessments to map residents’ pain, and these were used to complement the residents’ self-report. In addition, the nurses expressed how they must be constantly alert, even if the resident usually self-report:

We need to extract information. Even if they can answer a little themselves, and express themselves, we can’t just close our eyes. We must request it. He (a resident) shouted last night, and the nurse saw the blank eyes, that something was not right. And asked if he was in pain, ‘Yes!’ he shouted. Then she asked, ‘Where do you have pain? In the head?’ You must know what to look for: Someone else might have overlooked it.

Knowing the residents well constituted an essential aspect of the nurses’ clinical competence – having knowledge of the residents’ normal behaviour and of their lives enabled the nurses to give context and meaning to residents’ experience of pain. Thus, to make sense of the residents’ general expressions of pain, the nurses had to relate these to the context and what was normal for the resident, their history and other signs. The nurses depended on relational competence to distinguish between what was normal or what was an expression of something being wrong or ‘different’, requiring action. For instance, for one resident, wandering could signal a problem. For another resident, wandering could be normal – if that resident stopped wandering, however, this could be a sign that something was wrong.

### Overarching Theme: The Residents Express Themselves in the Moment and the Nurses Integrate These Moments into a Coherent Whole

Together, the two categories may be interpreted and united in an overarching, latent theme – ‘The residents express themselves in the moment and the nurses integrate these moments into a coherent whole’ – forming a complete picture of how pain narratives can be created in interactions between residents and nurses. This theme describes how (a) the residents often expressed themselves in the moment and (b) the nurses brought these expressions into a pain narrative consisting of several sources of information, putting fragments together to create an overall picture. The residents had individual ways of expressing their pain, influenced by their cognitive impairment, the origin of the pain, their personality, their knowledge of pain, their own previous experience with pain and the support they received from the nurses. The residents were dependent on support from someone (in this case, the nurses) with knowledge of time, space and their pain history – giving context and meaning to their expressions.

Relatives could also provide the nurses with information like a resident’s life story, pain history and how the residents had previously dealt with and expressed pain. Such information could help to explain behaviours and experiences related to pain expressions. Appropriate pain management depended on how the nurses interpreted, acted on and built on the pain expressions in pain management processes. A combination of clinical and relational competence, attitudes and motivations helped the nurses to identify and act when something differed from the normal and/or residents expressed pain. They subsequently discussed their observations and interpretations with colleagues, and the residents’ pain narrative was shared and further developed. These discussions were important, as they provided information about how to act in the future – and importantly, *what* to act on. While the pain narrative emerged in the interaction between the resident and the nurses, the nurses had the power to maintain the narrative by acting on it through pain management processes.

## Discussion

This study aimed to explore pain management in people with dementia living in nursing homes, focusing on the resources people with dementia have and how nurses can support and integrate these resources into their clinical practice. The findings add to the literature by demonstrating how the residents in this study often expressed themselves in the moment. The nurses then supported these expressions and brought them into a pain narrative consisting of several sources of information, piecing together fragments to form an overall picture. Our findings also underline the substantial individual variation in pain expression and perception that can be found in a nursing home unit, underpinning the complexity in pain management processes.

Study findings show the importance of grounding pain management in the resident’s perspective, when possible, and of seeking a holistic understanding of the pain experience – even in situations that are not necessarily physically painful. Palliative care emphasizes ‘total pain’, a multidimensional understanding of pain that includes physical, psychological, social, spiritual/existential and cultural aspects (Brant, 2017). With a palliative care approach to pain assessment that emphasizes total pain (van der Steen et al., 2014), there is great value in knowing the resident’s individual experience (recall, for example, the resident who fell out of bed and described it as lying under a fishing pier for hours). This knowledge might help to explain a resident’s behaviour and identify the support the resident needs, in the form of environmental measures. Both the residents and the nurses described a multidimensional understanding of pain, closely connected to total pain. We observed several repeated instances in which residents expressed an experience of physical pain – and then relief from that pain after environmental adjustments were made to increase their sense of security. This highlights the importance that healthcare personnel acknowledge and implement measures targeting the different dimensions of total pain. Pain management in nursing homes may involve various formal care providers, including not only nurses but also physicians, physiotherapists or occupational therapists. The capabilities of the residents are equally important when interacting with these care providers; therefore, we use the collective term ‘healthcare personnel’ in the following discussion.

Our overarching theme underscores how people with dementia rely on their expressions of pain being integrated into a larger overall picture of their pain symptoms. Due to cognitive challenges, residents’ expressions of pain must be contextualized with healthcare personnels’ assessments and observations. However, for people with dementia to be able to communicate their experience of pain effectively, they must be included; they need to be asked questions and supported in their communication, and healthcare personnel must seek out the essence of and meaning in residents’ responses – even if those responses do not correspond to what the healthcare personnel objectively perceive. To mitigate this, healthcare personnel should endeavour to assess residents’ pain in quiet, familiar and safe surroundings. Safe body language and adapted communication, such as speaking in short, concrete sentences or taking account of increased latency, can promote self-reporting of pain. These are all recognized communication methods in interacting with people in moderate- to severe stages of dementia (Alsawy et al., 2017).

The example of the fishing pier illustrates how embodied memory (Kontos, 2004) can be expressed in an experience of pain. Residents articulate their current situation by drawing on past experiences. This highlights how residents with dementia and healthcare personnel can occupy the same physical space without sharing the same perceived space (Eriksen et al., 2021). It emphasizes the importance of a person centred-care environment – where the residents’ voices are heard and they are supported to actively engage in relationships and interactions with healthcare personnel.

The results of our study advocate for a narrative care approach, encouraging care providers to support both the embodied and tell-able narrativity of pain in people with dementia (Berendonk et al., 2020). In our study, the pain narratives were created in the interactions between nurses and residents. This is supported by Berendonk et al. (2020) promoting care as a narrative practice in which healthcare personnel and care receivers cocreate narratives in their interactions. Berendonk et al. (2020) further describe how narrative care contributes to holding people with dementia in their stories, while simultaneously fostering the cocreation of new stories, akin to how our results portray nurses integrating pain expressions into a cohesive whole. One challenge with such an approach is the potential for differing perspectives on a situation between healthcare personnel (Bagchus et al., 2024); leading to disagreements among healthcare personnel regarding the narrative or the creation of multiple narratives. Berendonk et al. (2020) emphasize the importance of ongoing exchanges of stories and reflections on embodied narratives among healthcare personnel, as well as a systematic approach to sharing insights with the residents’ next of kins.

Our findings highlight how knowing the resident and relational continuity is a prerequisite for optimal pain management processes. Haggerty et al. (2003) describe disease- and person-focused information as equally important. However, person-focused information is typically accumulated in the memory of the providers who interact with the resident (Haggerty et al., 2003). While this knowledge is more intuitive and harder to articulate and communicate, it is essential for recognizing changes in nursing home residents (Bagchus et al., 2024). Further, effective pain management depends on action competence, requiring healthcare personnel to apply practical applications, such as assessments, decision-making and efficient measures (Nygren, 2004). Featherstone et al. (2019) highlights how healthcare personnel can misunderstand communication of pain in people with dementia as resistance to care. Combining knowledge about dementia and behavioural expressions as communication, with relational knowledge about the person, can help to reduce such misunderstandings. The nursing home as context is well placed to create such relational continuity (Rokstad et al., 2023).

### Strengths and Limitations

The study’s design allowed for an in-depth understanding of the complexity of pain management in people with dementia within a nursing home context. The inductive and flexible approach, utilizing several data sources, opened new perspectives and effectively targeted the study’s aim. The involvement of several researchers in the analysis constitutes an additional strength of the study. Our preunderstandings are also a strength, as they provided a foundation from which to understand the complex processes involved (Neumann & Neumann, 2023). Nevertheless, there is a potential risk of prejudice or that important aspects were overlooked (Wadel, 1991). To mitigate this risk, we distinguished between descriptive and reflexive field notes. We have also endeavoured to be transparent, supporting the presentation of our results with suitable quotes and detailed descriptions of relevant situations.

## Conclusion

There is significant individual variation in the pain expressions and perception of people with dementia living in nursing homes. These residents have individual resources and strengths that are important for pain management. Our findings highlight the potential of grounding pain management in residents’ perspectives when healthcare personnel interpret, communicate and act on their pain expressions, whether they arise from self-reporting or observed behaviour change. Healthcare personnel can utilize their knowledge of the resident´s life and medical history to provide context and meaning to the pain experience. Pain management places significant individual demands on healthcare personnel, highlighting the importance of organizational prerequisites needed. To facilitate the integration of resources for people with dementia into the pain management processes, nursing homes must promote coherence in healthcare services, the right expertise among healthcare personnel, and especially facilitate a pronounced person-centred culture that focuses on the resident’s perspective, preferences and own experience of the situation.

## Supplemental Material

Supplemental Material - Pain Management in People With Dementia Living in Nursing Homes: A Focused Ethnographic Study Supplemental Material for Pain Management in People With Dementia Living in Nursing Homes: A Focused Ethnographic Study by Caroline Kreppen Overen, Siren Eriksen, Maria Larsson, and Adelheid Hummelvoll Hillestad in Dementia

## Data Availability

As the data set encompasses confidential personal details, dissemination is not permissible.*
