# Supplementary material for: Pain Management in People With Dementia Living in Nursing Homes: A Focused Ethnographic Study
Source: Dementia (London). 2025 Jul 3;25(5):1039–56. doi: 10.1177/14713012251358003 (PMC13260738; doi:10.1177/14713012251358003)
Supplement: Supplemental Material - Pain Management in People With Dementia Living in Nursing Homes: A Focused Ethnographic Study [file sj-pdf-1-dem-10.1177_14713012251358003.pdf]

Supplementary file 1 Standards for reporting qualitative research

No.	Topic	Page(s)
	**Title and Abstract**	
1	Title	Title page
2	Abstract	1
	**Introduction**	
3	Problem formulation	2-3
4	Purpose or research question	3
	**Methods**	
5	Qualitative approach and research paradigm	4
6	Researchers' characteristics and reflexivity	7
7	Context	4
8	Sampling strategy	4-5
9	Ethical issues pertaining to human subjects	Title page, 7
10	Data collection methods	5-6
11	Data collection instruments and technologies	5-6
12	Units of study	4-5
13	Data processing	5-6
14	Data analysis	6-7
15	Techniques to enhance trustworthiness	19
	**Results**	
16	Synthesis and interpretation	8-16
17	Links to empirical data	8-16
	**Discussion**	
18	Integration to prior work, implication, transferability, and contribution(s) to the field	16-19
19	Limitations	19
	**Other**	
20	Conflicts of interests	Title page
21	Funding	Title page

Reference:

O'Brien BC, Harris IB, Beckman TJ, et al. Standards for reporting qualitative research: A synthesis of recommendations. *Acad Med.* 2014;89:1245–51.
